# Acute pancreatitis with giant pancreatic pseudocyst as a complication of extracorporeal shock wave lithotripsy: A case report and literature review

**DOI:** 10.1016/j.amsu.2021.102442

**Published:** 2021-05-26

**Authors:** Hussam I.A. Alzeerelhouseini, Yousef S. Abuzneid, Hazem A. Ashhab

**Affiliations:** a Al-Quds University, Faculty of Medicine, Jerusalem, Palestine; b Al-Ahli Hospital, Hebron, Palestine

**Keywords:** ESWL, Stone, Pancreatitis, Pseudocyst, Stent, Case report, ESWL, Extracorporeal shock wave lithotripsy, PP, Pancreatic pseudocyst, EUS, Endoscopic ultrasound, LAMS, lumen-apposing metal stent

## Abstract

**Introduction:**

Extracorporeal shock wave lithotripsy has been confirmed as the least invasive and the most widely used treatment for kidney and ureteral stones. However, as with any other type of therapy, potential complications do exist.

**Case presentation:**

Herein, we describe a 55-year-old male patient who developed symptoms of acute pancreatitis one day after extracorporeal shock wave lithotripsy for left renal stones. The patient used to manage himself with NSAIDs till he presented to the emergency department with severe epigastric pain and tenderness due to giant pancreatic pseudocyst formation. The pseudocyst was treated by endoscopic cystogastrostomy using metallic stent with uneventful recovery. In addition, we extensively reviewed all available literature studies of pancreatitis and pancreatic pseudocyst occurring after extracorporeal shock wave lithotripsy. We summarized all reported cases and presented them in a comprehensive table.

**Discussion:**

Post ESWL acute pancreatitis is a rare clinical entity with only 11 reported cases. In all cases, abdominal pain was the most common symptom that occurs in less than 24h following ESWL treatment. So it should be considered in the differential diagnosis of acute abdominal pain after ESWL.

**Conclusion:**

Although ESWL is generally considered safe and effective treatment; however, major complications have been reported to occur in less than 1% of patients. One of the extremely rare complications is the development of pancreatitis and pancreatic pseudocyst.

## Highlights

• ESWL is a safe and effective treatment modality.
• Serious complications have been reported in 1% of patients.
• Post-ESWL acute pancreatitis is extremely rare.
• Only 5 cases of pancreatic pseudocyst after ESWL have been reported.
• Endoscopic cystogastrostomy approach is cost-saving, can avoid surgical complications, and offer an early hospital discharge.

## 1 Introduction

Extracorporeal shock wave lithotripsy (ESWL) is one of the most common urological procedures performed today and it is considered the treatment of choice for renal and upper ureteral stones [1]. ESWL is an effective and relatively safe modality for stone fragmentation with complication rates are typically low but can vary from those with minimal consequence to life-threatening injuries [2]. One of the strongly rare complications is the development of pancreatitis and pancreatic pseudocyst.

Pancreatic pseudocyst (PP) is a peripancreatic fluid collection that usually develops and matures 4–6 weeks after an episode of chronic pancreatitis, acute pancreatitis, or disruption in the pancreatic duct such as blunt, penetrating trauma, or injury during surgeries. It is mainly characterized by a well-defined inflammatory wall with a homogeneous fluid content that rich in amylase enzyme in the absence of tissue necrosis. A pseudocyst with a major diameter equal to 10 cm or more is called a giant pseudocyst [3].

Development of acute pancreatitis after ESWL is an extremely rare condition with only 11 cases reported in our literature, out of which only 5 cases developed into pancreatic pseudocyst Table 1. Herein we describe a unique case of acute pancreatitis complicated by giant pancreatic pseudocyst as a rare complication of extracorporeal shock wave lithotripsy for left kidney stones. The pseudocyst was treated by endoscopic cystogastrostomy using a lumen-apposing metal stent with uneventful recovery. The work has been reported in line with the SCARE 2020 criteria [14].

**Table 1:** Summarized clinical data of all published cases of pancreatitis after ESWL.

Case	Author, Year	Age	Sex	Stones location	Stone size (mm)	number of ESWL sessions	Energy (kv)/shocks	Presentations of pancreatitis	Onset time	laboratory investigation	Diagnosis	Pancreatic pseudocyst	Treatment
1	Abe et al., 2000 [4]	67 yr.	F	Right and left kidneys	11 × 6 (R) 13 × 10 (L)	Twice	20/2000 (1st) 20/2400 (R) 20/1400 (L) (2nd)	Upper abdominal pain and distension, back pain.	Within hours	Elevations of blood and urine amylase	necrotizing pancreatitis, necrosis of mesentery and omentum	No	Laparotomy with necrosectomy.
2	Hassan et al., 2002 [5]	43 yr.	M	Right kidney	5 × 8	Once	19/4000	Epigastric pain	8 hours	Elevated serum amylase and lipase	Acute pancreatitis	Yes	Acute pancreatitis: Conservative management. Pseudocyst: spontaneous regression.
3	Florio et al., 2003 [6]	39 yr.	M	Right kidney	Not reported	Once	Not reported	Asymptomatic	–	–	Pancreatic pseudocyst	Yes	Surgery (proximal pancreaticoduodenectomy).
4	Karakayali et al., 2006 [7]	39 yr.	M	Right kidney	4 × 2	Once	15/3500	Abdominal pain and distension, back pain.	6 hours	Elevated WBC, serum amylase and lipase	Necrotizing pancreatitis	Yes	Pancreatitis: parenteral nutrition and antibiotic. Pseudocyst: percutaneous drainage (pigtail catheter).
5	Hama et al., 2010 [8]	62 yr.	M	Right kidney	Not reported	Once	Not reported	Abdominal pain	Within hours	Elevated serum amylase	Acute pancreatitis	No	Conservative management.
6	Weng et al., 2013 [9]	57 yr.	M	Right and left kidneys	16 × 9 (R) 12 × 9 (L)	Four times	18–24/3000	Vomiting and abdominal pain	4 hours	Elevated WBC, serum amylase	necrotizing pancreatitis, peripancreatic abscess	No	Laparotomy (necrosectomy and drainage of peri-pancreatic abscess)
7	Limon et al., 2014 [10]	41 yr.	F	Right kidney	8.7	Three times	15/3002	Epigastric pain and tenderness	During the procedure.	Elevated WBC, serum amylase and lipase	Acute pancreatitis	No	Conservative management.
8	Mylarappa et al., 2014 [11]	21 yr.	M	Left kidney	10 × 8	Once	15/2700	Epigastric pain and distension, persistent vomiting	24 hours	Elevated WBC, serum amylase and lipase	Acute pancreatitis	Yes	Pancreatitis: conservative management. Pseudocyst: surgical drainage.
9	Goral et al., 2015 [2]	41 yr.	F	Right kidney	7	Three times	6-20/3000	Epigastric pain and tenderness, nausea, vomiting.	Within hours	Elevated WBC, serum amylase and lipase	Acute pancreatitis	No	Conservative management.
10	Gupta et al., 2016 [12]	29 yr.	F	Right kidney	11	Once	Not reported	Abdominal pain radiating to the back, epigastric tenderness, vomiting.	6 hours	Elevated WBC, serum amylase and lipase	Necrotizing pancreatitis	No	Laparotomy.
11	Randhawa et al., 2018 [13]	56 yr.	M	Left kidney	13	Once	15/3000	Epigastric pain and tenderness.	24 hours	Elevated serum amylase	Acute pancreatitis	Yes	Conservative management.
12	Alzeerelhouseini et al., 2021.	55 yr.	M	Left kidney	12	Once	15/3000	Epigastric pain radiating to the back	24 hours	–	Acute pancreatitis	Yes	Pseudocyst: endoscopic cystogastrostomy using lumen-apposing metal stent (SPAXUS).

## 2 Case presentation

A 55-year-old male was admitted to our hospital complaining of intermittent left-sided abdominal pain for two weeks. Vital signs were within normal and examination was unremarkable except for mild abdominal tenderness in the left upper quadrant. Urine analysis revealed mild hematuria, and abdominal ultrasound was performed which showed two stones in the lower pole of the left kidney measuring about 4 and 12 mm with an absence of hydronephrosis and normal right kidney. As a treatment for his condition, extracorporeal shock wave lithotripsy (ESWL) was performed with a total of 3,000 shocks were delivered at 15 kv. The patient tolerated the procedure well, and there were no complications during the procedure.

**Fig. 1:**
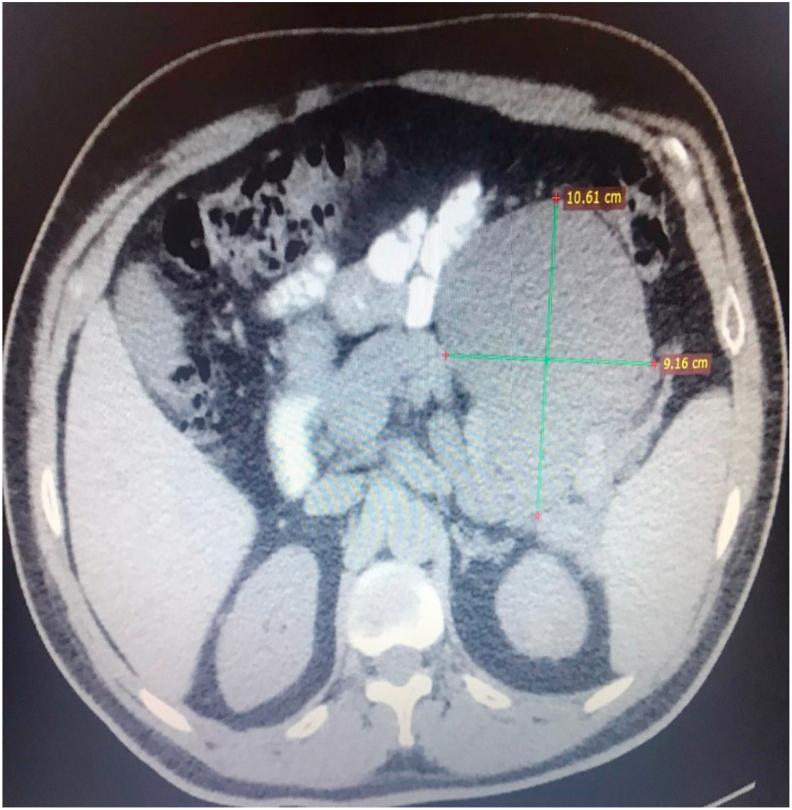
Abdominal CT scan showing a huge pancreatic pseudocyst occupying the body and tail of the pancreas.

One day after ESWL, the patient started complaining of mild to moderate epigastric pain radiating to the back, the pain decreased with ibuprofen administration and the patient didn't seek medical advice. About seven weeks after ESWL, the patient presented to the emergency department with severe epigastric pain associated with nausea, vomiting, and early satiety. The patient had free past medical and surgical history and he is off medication and no history of alcohol consumption. On general examination, he was not icteric or feverish and an abdominal exam revealed epigastric fullness with severe tenderness. Laboratory investigations revealed markedly elevated serum amylase (6320 U/L). Complete blood count (CBC), liver enzymes, serum electrolytes, and lipid profile were within normal limits. An abdominal CT scan with contrast showed a giant multiseptated cyst measuring about 10 × 11 cm occupying the body and the tail of the pancreas (Fig. 1). Few small stones in the lower calyx of the left kidney also were found. The gallbladder, intrahepatic and extrahepatic biliary systems were completely normal.

**Fig. 2:**
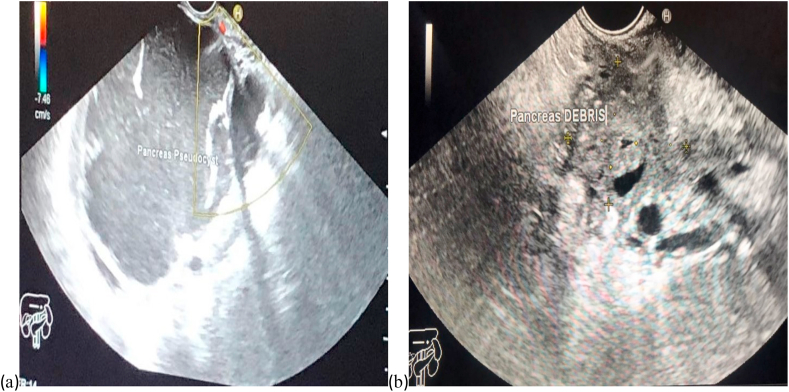
EUS image showing (a) a pancreatic pseudocyst before drainage and (b) pseudocyst after drainage.

**Fig. 3:**
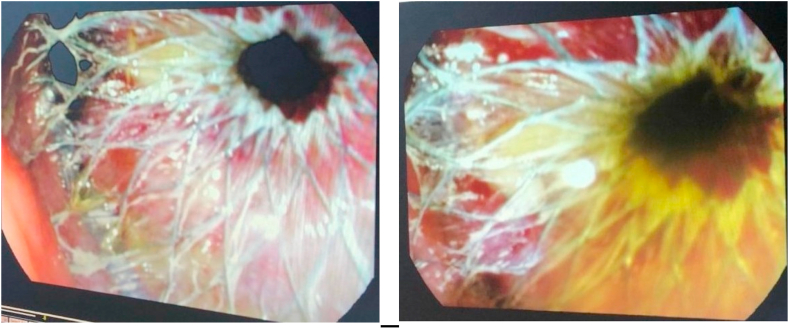
Endoscopic view showing SPAXUS stent.

Endoscopic ultrasound- (EUS-) guided drainage was performed as a therapeutic procedure for giant symptomatic pseudocyst using a Pentax linear echoendoscope (EG-3870UTK 3.8). On the EUS, there was a huge pancreatic pseudocyst with internal septation (Fig. 2 (a)). The Niti-S SPAXUS stent (from Taewoong Medical) with 16 mm diameter, 20 mm length was placed with excellent drainage of >1500 cc of fluid and debris (Fig. 3). The patient tolerated the procedure well, and there were no complications. One day after the procedure, a CT scan showed excellent results with regression of the pseudocyst, and the patient was discharged home on levofloxacin 750 mg for five days.

On 4 weeks of follow-up, the patient reported disappearance of symptoms. Repeated EUS showed resolution of the pseudocyst and SPAXUS stent was removed without complications (Fig. 2 (b)).

## 3 Discussion

Extracorporeal shock wave lithotripsy (ESWL) has been widely used for the treatment of upper urinary tract stones since it was first introduced in 1980. Also, it is sometimes used in the treatment of pancreatic and bile duct stones [11]. ESWL is an effective, non-invasive treatment with mild complications that occur in less than 10% of the patients which can include transient gross hematuria, flank pain, and urinary tract infection that can usually be treated with conservative management [15].

Adjacent organ injury was reported to occur in less than 1% of patients undergoing ESWL procedure with serious complications include perirenal hematoma, urosepsis, pulmonary contusion, new-onset diabetes mellitus, cardiac arrhythmia, rupture of aortic aneurysm, colonic and splenic injury, venous thrombosis, and severe acute pancreatitis that may lead to pancreatic pseudocyst formation [7].

Multiple theories describe how ESWL can cause acute pancreatitis and other tissue damage. One theory proposes that cellular damage caused by cavitation and shear forces produced by the passage of shock waves as they pass through the body may cause pancreatic injury after ESWL. These shock waves may cause hematoma formation and microvascular pancreatic damage [5]. Another theory suggests that direct injury can result in adhesion formation between tissues which can worsen symptoms. It has also been suggested that infected urine leakage secondary to renal injury during ESWL could precipitate pancreatitis [4]. Inadvertent fragmentation of gallstones or common bile duct stones may also cause pancreatic duct obstruction leading to acute biliary pancreatitis [16]. However, the last two theories don't match with our case since no biliary stone was demonstrated on radiologic studies and the results of urinalysis and urine culture were normal.

Some studies suggest that the delivered number and intensity of shock waves could be a possible etiological factor [5]. According to our literature, although there are cases of pancreatitis that occur after three and four sessions of ESWL, and the case that was reported by Abe had severe necrotizing pancreatitis with necrosis of mesentery and omentum after two sessions of ESWL for each kidney. However, in most cases, acute pancreatitis developed after the first session of ESWL and with a standard intensity level Table 1.

Although the exact mechanism of pancreatitis after ESWL is still unclear. However, the absence of any of the predisposing factors such as gall stone, alcohol, hypercalcemia, hypertriglyceridemia, and prior abdomen surgery, support that ESWL is the cause of pancreatitis in our case.

Table 1 summarizes the characteristics of all published cases of pancreatitis after ESWL. Lithotripsy was performed on renal stones in all cases, with a proportion of pancreatitis occurring more in males with a male to female ratio equal to 2:1. Also, we can see that pancreatitis occurred in 7 patients following ESWL to a right renal calculus, 2 cases following lithotripsy to bilateral renal calculi, and 3 cases following lithotripsy to left renal calculus (including our case). Moreover, we noted that in all reported cases the symptoms of acute pancreatitis started in less than 24h after ESWL procedure with epigastric pain is the most characteristic symptom. Besides, leukocytosis and elevated serum amylase and/or lipase are the most prominent laboratory investigations. Hence, epigastric pain occurring after ESWL should increase the suspicion of acute pancreatitis.

The course of acute pancreatitis following ESWL is variable, most of the patients resolved with conservative management that includes nasogastric tube insertion, bowel rest, parenteral nutrition, intravenous antibiotics. However, some can develop necrotizing pancreatitis requiring necrosectomy, while others developed pseudocysts as in our case Table 1. The management decision of pancreatic pseudocyst mainly depends on clinical and imaging evaluation. Most of the pseudocysts are asymptomatic and observation till spontaneous regression is all what is required. However, symptomatic, persistent, large, or complicated pseudocysts require internal drainage surgically or by using less-invasive percutaneous or endoscopic approaches [17]. Endoscopic drainage of pancreatic pseudocyst by creation of a fistulous tract between the pseudocyst and the gastric lumen (cystogastrostomy) is an alternative non-surgical approach for pancreatic pseudocysts’ management that lately gained a wide range of acceptance due to its lower morbidity, mortality, and costs [3].

Traditionally, EUS-guided drainage could only be performed using plastic stents like double pigtail stent (DPS) or a fully covered self-expanding metal stent. Recently, a dedicated device, a lumen-apposing metal stent (LAMS), has been developed as an alternative for pancreatic fluid collection. Lumen-apposing metal stents like AXIOS and Niti-S SPAXUS stents with their large diameter, short length, and bi-flanged shape have less risk of stent migration, perforations, leakage, occlusion, and superimposed infection as compared with the plastic stent [18]. Moreover, LAMSs have become the stent of choice for endoscopic drainage of pancreatic fluid collection by many gastroenterologists, because of their easy deployment, less procedure time, and direct debridement access [19].

The main drawback of LAMS that it was found to be associated with a higher bleeding rate when compared to the plastic stent, including late bleeding at 3–5 weeks after stent insertion which is mainly caused by stent friction to surrounding vasculature around the necrotic cavity promoting pseudoaneurysm formation and subsequent bleeding. This prompted gastroenterologists to change their practice of repeating imaging at 3 weeks to assess the cavity resolution instead of 6 weeks, followed by stent removal if the fluid collection is resolved or placing plastic double pigtail stent inside the LAMS [3].

## 4 Conclusion

ESWL is a safe, effective, and non-invasive procedure; However, serious complications to adjacent organs rarely can occur. Acute pancreatitis should be considered as a differential diagnosis in patients presenting with acute abdominal pain following extracorporeal shock wave lithotripsy, as prompt diagnosis and essential treatment can prevent significant morbidity and death.

## Ethical approval

The study is exempt from ethical approval in our institution.

## Sources of funding

This research did not receive any specific grant from funding agencies in the public, commercial, or not-for-profit sectors.

## Authors’ contributions

Hazem A. Ashhab diagnosed and treated the patient. Hussam I. A. Alzeerelhouseini, Yousef S. Abuzneid: Study design and writing the manuscript. Hazem A. Ashhab, Hussam I. A. Alzeerelhouseini: Writing the literature, Review & editing the manuscript.

## Guarantor

Dr. Hussam I. A. Alzeerelhouseini.

## Consent

Written informed consent was obtained from the patient for publication of this case report and accompanying images. A copy of the written consent is available for review by the Editor-in-Chief of this journal on request.

## Declaration of competing interest

There is no conflict of interest.
